# Novel aspects in the regulation of follicular development and ovulation rate: forum introduction

**DOI:** 10.1186/1477-7827-4-15

**Published:** 2006-04-06

**Authors:** Jennifer L Juengel

**Affiliations:** 1 Forum Coordinator, AgResearch Limited, Wallaceville Animal Research Centre, Ward Street, PO Box 40063, Upper Hutt, New Zealand

It is well known that the control of follicular growth and development involves a complex exchange of signals between the pituitary gland and the ovary and among ovarian cells themselves. Although the roles for some hormones in regulating follicular growth are well established, recent research has led to the identification of new hormones and growth factors that regulate follicular development and ovulation rate, and to redefining the roles of some of the classic reproductive hormones. The purpose of this forum is to provide an update on the roles of some of these novel factors and as well some of the classic factors in regulating ovarian follicular development.

The forum begins with a review by A Drummond regarding the role of steroids in ovarian follicular development, a classic hormone in which we are continuing to discover novel actions for. This is followed by a review by Abbott et al. who look at the consequences of an inappropriate exposure to steroids on the developing ovary and by H Fraser, who details the regulation of the development of the follicular vasculature. Next is a review by Thomas and Vanderhyden, who explore the interactions between the oocyte and the cumulus cells and the factors that regulate and are regulated by this communication. Following this is an article by Fabre et al., who discuss the use of sheep carrying single genetic mutation as models to elucidate mechanisms regulating ovulation rate. The next review by Kaivo-oja et al. covers the function of the Smad proteins, the signalling molecules for the transforming growth factor beta (TGFbeta) superfamily, in regulation of ovarian function. The final article by Kobayashi et al., investigates the concept that follicles may be selected for atresia as they are selected for ovulation and discusses the factors that may be involved in this "negative" selection.

